# Supplementary material for: A learned score function improves the power of mass spectrometry database search
Source: Bioinformatics. 2024 Jun 28;40(Suppl 1):i410–7. doi: 10.1093/bioinformatics/btae218 (PMC11211853; doi:10.1093/bioinformatics/btae218)
Supplement: btae218_Supplementary_Data [file btae218_supplementary_data.zip › btae218_Supplementary_Data/casanovo-db-supplement.pdf]

Supplement to "A learned score function improves the power of mass spectrometry database search"

Varun Ananth*[1], Justin Sanders*[1], Melih Yilmaz[1], Bo Wen[2], Sewoong Oh[1], and William Stafford Noble[2,1]

[1]Paul G. Allen School of Computer Science and Engineering, University of Washington
[2]Department of Genome Sciences, University of Washington

*Equal contributions

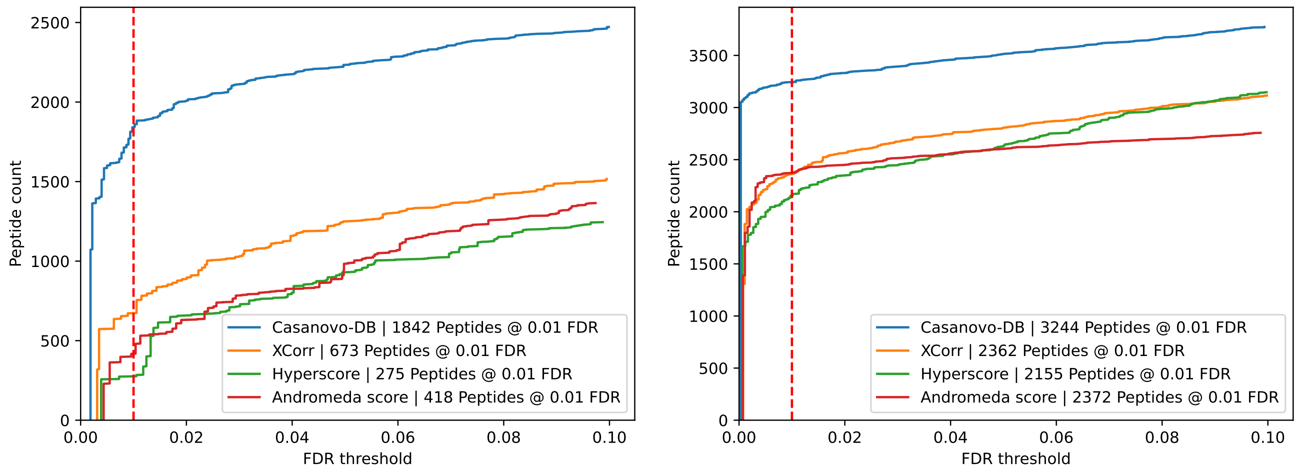

(a) (b)

Figure S1: Each figure plots the number of peptides detected as a function of FDR threshold for the *E. coli* dataset, broken down by whether the precursor had an m/z in (a) the bottom quartile range of 350–467 m/z or (b) the top quartile range of 704–1389 m/z. In each plot, the series correspond to different score functions, each followed by Percolator post-processing, and the 1% FDR threshold is highlighted with a red dashed line.

	All four		Not Casanovo-DB		Only Casanovo-DB	
	All	C-term	All	C-term	All	C-term
A	8.13	0.04	10.81	0.00	5.95	0.03
C	1.50	0.00	1.40	1.85	1.20	0.00
D	6.55	0.07	4.20	0.00	5.51	0.04
E	9.40	0.10	9.61	0.00	8.10	0.03
F	3.27	0.02	2.30	1.85	4.85	0.04
G	7.28	0.01	7.11	0.00	5.32	0.03
H	1.89	0.06	3.20	0.00	2.08	0.03
I	5.09	0.02	5.21	1.85	6.01	0.01
K	3.91	53.86	3.10	50.00	6.90	59.44
L	9.43	0.04	9.11	0.00	12.36	0.12
M	1.63	0.00	2.70	0.00	2.24	0.01
N	4.36	0.07	5.11	0.00	3.69	0.01
P	5.76	0.02	7.51	0.00	5.58	0.00
Q	5.26	0.04	6.71	0.00	4.85	0.06
R	3.31	45.48	2.80	42.59	4.74	39.99
S	7.06	0.03	6.01	0.00	5.79	0.04
T	5.81	0.01	4.90	0.00	4.53	0.03
V	7.25	0.06	6.31	0.00	6.52	0.04
W	0.50	0.00	0.30	0.00	0.93	0.00
Y	2.63	0.04	1.60	1.85	2.83	0.03
Count	13,495		54		9442	
Mean length	15.3		18.5		9.3	
% modified	11.4		37.1		22.3	

Table S1: **Comparison of subsets of detected PTMs.** The table lists various statistics—overall amino acid frequencies, amino acid frequencies at the C-terminus, total number, mean peptide length, and percentage of peptides that contain a variable modification—for four sets of peptides: those detected from the human dataset by (1) all four search methods, (2) all three methods except Casanovo-DB, and (3) Casanovo-DB only.
